# Changes in Brain-Health Related Modifiable Risk Factors in Older Adults After One Year of COVID-19-Restrictions

**DOI:** 10.3389/fpsyt.2022.877460

**Published:** 2022-06-02

**Authors:** Lisa Waterink, Els D. Bakker, Leonie N. C. Visser, Francesca Mangialasche, Miia Kivipelto, Kay Deckers, Sebastian Köhler, Sietske A. M. Sikkes, Niels D. Prins, Philip Scheltens, Wiesje M. van der Flier, Marissa D. Zwan

**Affiliations:** ^1^Alzheimer Center Amsterdam, Amsterdam University Medical Center, Amsterdam Neuroscience, Department of Neurology, Vrije Universiteit Amsterdam, Amsterdam, Netherlands; ^2^Division of Clinical Geriatrics, Center for Alzheimer Research, Department of Neurobiology, Care Sciences and Society, Karolinska Institutet, Stockholm, Sweden; ^3^Department of Medical Psychology, Amsterdam Public Health Research Institute, Amsterdam UMC Location AMC, Amsterdam, Netherlands; ^4^Ageing Epidemiology Research Unit, School of Public Health, Imperial College London, London, United Kingdom; ^5^Institute of Public Health and Clinical Nutrition, University of Eastern Finland, Kuopio, Finland; ^6^Medical Unit Aging, Theme Inflammation and Aging, Karolinska University Hospital, Stockholm, Sweden; ^7^Alzheimer Center Limburg, School for Mental Health and Neuroscience, Maastricht University, Maastricht, Netherlands; ^8^Faculty of Behavioural and Movement Sciences, Department of Clinical, Neuro and Developmental Psychology, Vrije Universiteit, Amsterdam, Netherlands; ^9^Brain Research Center, Amsterdam, Netherlands; ^10^Life Sciences Partners Dementia Fund, Amsterdam, Netherlands; ^11^Department of Epidemiology and Data Science, Amsterdam University Medical Center, Vrije Universiteit Amsterdam, Amsterdam, Netherlands

**Keywords:** COVID-19, lifestyle, mental health, aging, risk factors, cognitive decline, prevention, lockdown

## Abstract

**Background:**

The COVID-19 pandemic has major influence on lifestyle and mental health, which might affect brain-health and increase the risk of cognitive decline, particularly in older adults. We aimed to describe changes in modifiable risk factors related to brain-health in older adults after one year of COVID-19 restrictions.

**Methods:**

An online survey was disseminated between February and March 2021 to 17,773 registrants of the Dutch Brain Research Registry, aged ≥50, without a self-reported diagnosis of mild cognitive impairment or dementia. Participants were asked to report potential changes in behaviors during the COVID-19 pandemic, compared to pre-pandemic, in eight domains related to brain health: physical activity, sleep, feeling of memory decline, perceived stress, feeling of loneliness, diet, alcohol consumption, and smoking. We used negative binomial regression analyses to relate (socio)demographics, subjective memory complaints and COVID-19 related aspects (fear of, or current/past COVID-19 infection) to the number of reported detrimental and beneficial changes as dependent variable.

**Results:**

3,943 participants (66 ± 8 years old; 76% female; 71% highly educated) completed the survey. After one year of COVID-19-restrictions, 74% reported at least one detrimental lifestyle change unfavorable for their brain health, most frequently reported were feelings of loneliness, sleep problems, and less physical activity. 60% of participants reported at least one beneficial change, which were most often more physical activity, healthier dietary habits, and less alcohol consumption. Individuals who are younger [incidence rate ratio (IRR) = 0.99, 95% CI = 0.98–0.99], female (1.20, 1.11–1.30), living alone (1.20, 1.11–1.28) and in urban environments (1.18, 1.08–1.29), who are less satisfied with their income (1.38, 1.17–1.62), experiencing subjective memory complaints (1.40, 1.28–1.52) and those with a past or current (1.19, 1.06–1.34) or fear of a COVID-19 infection (1.33, 1.25–1.42) reported higher numbers of detrimental changes.

**Discussion:**

The COVID-19 pandemic has influenced lifestyle in both positive and negative ways. We identified (socio)demographic factors associated with more detrimental changes in modifiable risk factors related to brain health, suggesting that some individuals are more vulnerable for the impact of the COVID-19 pandemic. These findings provide an opportunity for targeted prevention and education to promote a healthy lifestyle during and after the pandemic.

## Introduction

The coronavirus disease 2019 (COVID-19) pandemic has, due to public health recommendations and governmental measures, resulted in the closing of social, cultural and sports facilities and many restrictions on daily living, including isolation, social distancing, and home confinement. The pandemic and related restrictions have been reported to impact lifestyle and mental health in the general population (1, 2). In older individuals particularly, enduring unhealthy changes in lifestyle and mental health may affect brain-health, potentially altering the risk for accelerated cognitive decline. As such, serious concerns exist about the impact of the COVID-19 pandemic and related restrictions on brain-health (3). Since 40% of dementia cases are potentially attributable to modifiable factors (e.g., physical inactivity, depression, social isolation and smoking) (4), and evidence about effective prevention of cognitive decline with multi-domain lifestyle interventions is emerging (5–7), knowledge about the impact of the COVID-19 pandemic on brain-health related risk factors is important for the prevention of accelerated cognitive decline (8).

Throughout the pandemic, many countries experienced multiple “waves” in which infections and hospitalizations due to COVID-19 increased, resulting in constant adjustment of recommendations and governmental measures. In the Netherlands, the first wave was from March 2020 till mid May 2020, and the second wave was from October 2020 till February 2021 when additional to other restrictions a curfew was imposed. In March 2021 the number of infections rose again and a third wave made its entry which lasted until the end of April 2021. Surveys conducted during the first waves across multiple countries showed that the COVID-19 restrictions affected lifestyle behaviors, for example, decreases in physical activity (2, 9–12), more sleep problems (2, 9, 13), and increase in stress-related feelings (14) were repeatedly reported. Some studies showed that alcohol binge drinking and smoking decreased (15, 16) or remained unchanged (9) during the lockdowns while others reported an increase in alcohol consumption and smoking (2, 12), and several studies also reported changes in dietary habits (the type of food, snacks between meals, and number of main meals) (12, 17). Furthermore, during the first wave, an increase in subjective memory complaints was observed (14–37%) (13, 14, 18), which may be first signs of cognitive decline (19, 20). This emphasizes the importance of knowledge about lifestyle changes during the COVID-19 pandemic in the context of cognitive decline and brain health. In addition to detrimental lifestyle changes, favorable lifestyle changes have also been reported, although by a smaller proportion of participants (9, 12, 17), and in a large Dutch and Finnish sample, the majority of respondents reported no change in various lifestyle behaviors as a reaction to COVID-19 restrictions (9, 21). Conflicting findings may be due to differences in governmental measures, and for example if these measures were forced by law or appealed to the responsibility of the citizens. Also, social and cultural differences across countries may influence behavioral reactions to the COVID-19 restrictions. Given this mixture of findings and ongoing COVID-19 pandemic with related restrictions, insight in the long-term effects on lifestyle changes in different countries is needed.

Therefore, the World-Wide-FINGERS-SARS-CoV-2 survey was developed to assess changes in lifestyle and psychosocial factors as a result of the COVID-19 restrictions (9) within the context of the World-Wide FINGERS (WW-FINGERS) network of multi-domain prevention trials^1^ (22, 23). We used this survey to explore which changes in modifiable risk factors related to brain-health occurred among older adults in the Netherlands during the first year of the COVID-19 pandemic which included the first and second wave (March 2020–2021). Our primary goal was to improve the knowledge of lifestyle changes due to COVID-19 restrictions. Campaigns throughout the pandemic primarily focused on recommendations to fight the spread of infections, with limited focus on healthy lifestyle. Our results may contribute to designing health education campaigns to promote a healthy lifestyle during and after pandemics. Secondly, we aimed to identify which participants’ (socio)demographics and COVID-19-related factors were associated with detrimental or beneficial changes in lifestyle. Identification of individuals with increased risk of future cognitive decline is important for targeted dissemination of health education and selection of potential candidates for multi-domain lifestyle interventions.

## Materials and Methods

### Participants and Procedures

For this population-based cross-sectional study we recruited participants via the Dutch Brain Research Registry, a nationwide online platform for people interested in participating in brain-related research (24). From the Dutch Brain Research Registry, 17,773 registrants received a study invitation per email, of which 3,943 completed the online survey and were included in the study. Registrants aged 50 years and older and without a self-reported diagnosis of mild cognitive impairment or dementia were included. The online survey was offered from February 2021 till March 2021 and consisted of questions about lifestyle and mental health (9) and questions about psychosocial factors (25) (Supplementary Table 1). Information on (socio)demographics, subjective memory complaints including worries prior the COVID-19 pandemic and “LIfestyle for BRAin health” (LIBRA) score were collected from the Dutch Brain Research Registry.

### Measures

#### (Socio)demographics and Subjective Memory Complaints

As (socio)demographic factors we included age, sex, education, professional status (unemployed, employed, or retired), subjective measure of financial situation (unsatisfactory, satisfactory or more than satisfactory), living alone (yes/no), population density (rural, urban <40.000 inhabitants, urban: >40.000 inhabitants). We dichotomized education level into low–medium (up to the equivalent of high school education) and high education (the equivalent of college education or higher). Presence of subjective memory complaints was defined as presence of complaints (yes/no) and worries about this (yes/no).

#### Individual’s Health and Lifestyle Risk for Cognitive Decline

Information about modifiable health and lifestyle risk factors for cognitive decline and dementia was included, to provide an indication of an individual’s potential for dementia risk reduction. For this we calculated the LIBRA, which is a validated risk score developed after triangulation of results from a systematic literature review and an expert consensus study (26, 27). Risk factors are coronary heart disease, diabetes, hypercholesterolemia, hypertension, depression, obesity, smoking, physical inactivity, and renal disease. Protective factors are a healthy diet (Mediterranean), cognitively active and low-to-moderate alcohol intake. The cut-off for low-to-moderate alcohol consumption is based on the Dutch Dietary Guidelines, which states that alcohol consumption should be avoided (no drinks) or not more than one drink per day (28). Based on a weighted sum score of nine risk factors and three protective factors (theoretical range from −5.9 to +12.7; with higher scores indicating greater risk of cognitive decline or dementia; see Supplementary Table 2), which were available for half of our participants (1,984/3,943, 50%).

#### Changes in Modifiable Factors Related to Brain Health During the COVID-19 Pandemic

We described changes based on eight modifiable factors related to brain health: physical activity, diet, alcohol consumption, smoking, feeling of memory decline, sleep, perceived stress, and loneliness (Supplementary Table 1). Participants were asked to indicate an increase or decrease on each of these domains, compared to before the COVID-19-outbreak, either on a three- or five-point scale. Questions formulated as a five-point scale were merged into a three-point scale (“decreased/increased a little” merged with “decreased/increased a lot,” “clearly worse/better” merged with “slightly worse/better”), and coded as a minus one “detrimental change,” plus one “beneficial change” and zero “no change” (Supplementary Table 1). For all questions, “does not concern me” was categorized as “no change.” Questions for which a decrease was considered as beneficial (i.e., loneliness, sleep problems, unhealthy snacks, alcohol consumption and smoking) were reversely scored for ease of interpretation. If multiple items in the survey covered the same factor, these were averaged. For physical activity, the change in amount of leisure sport activities during COVID-19 pandemic and before was calculated from two separate questions (Supplementary Table 1). If participants reported to have a (sport) injury before (*n* = 97, 2.5%) or during (*n* = 121, 3.1%) the COVID-19 pandemic this was considered as “no change” since change was clearly not due to the COVID-19 restrictions. For each participant, we counted the number of detrimental and the number of beneficial factors (both; range 0–8).

#### COVID Related Factors

As COVID-19-related factors we included two questions, if the participant experienced current or past COVID-19 infection (yes/no) or had fear of getting infected with COVID-19 (yes/no).

### Statistical Analyses

Descriptive statistics include absolute frequencies and percentages for categorical variables, and mean ± standard deviation for continuous variables. Since our outcomes were count variables, we used multiple negative binomial regression analyses to explore associations between participant characteristics and the number of detrimental and beneficial changes (separate models) on modifiable factors related to brain-health during the COVID-19 outbreak. Participant characteristics included (socio)demographics, presence of subjective memory complaints and worries, the individual’s LIBRA score, current/previous COVID-19-related infection, and fear of COVID-19 infection.

First, we performed univariate analyses for each predictor separately, adjusted for age and sex (Model 1). Secondly, all predictors were evaluated simultaneously in a multivariable model to estimate independent statistical associations (Model 2). Out of the total sample (*n* = 3943), 3274 participants (83.0%) provided response for all variables of interest and were included in the final model. We repeated the analysis in the subsample with available LIBRA score, where LIBRA score was evaluated as additional determinant set of analyses (*n* = 1984, 50.3%). In order to correct for the effect on lifestyle potentially caused by the illness due to a COVID-19 infection, we performed a sensitivity analysis in which we excluded participants who reported to have a past/current COVID-19-infection (*n* = 252, 6.4%). Estimates are presented as incidence rate ratio’s (IRR) and their 95% confidence intervals (CI). The level of statistical significance was *p* < 0.05 in two-sided tests, due to the exploratory approach we did not correct for multiple testing. All analyses were performed in SPSS Statistics version 26.

## Results

### Participant Characteristics

Participants were aged between 50 and 99 years (mean ± SD = 66 ± 8), the majority was female (*n* = 2988, 75.8%) and highly educated (*n* = 2799, 71.0%). Table 1 shows participants’ (socio)demographics including frequencies of self-reported risk and protective factors as measured by the LIBRA. The LIBRA score ranged from −5.9 to 7.8, with an average of −0.32 (±2.2) indicating a relatively healthy sample with generally more protective factors for cognitive decline (29). For the COVID-19 related aspects, 252 participants (6.4%) reported a current or past COVID-19 infection, and 1248 (31.7%) expressed fear of getting infected with COVID-19.

**TABLE 1:** Participant characteristics.

	Total (*n* = 3943)
Female	2,988 (75.8%)
Age in years	66 ± 8
Education^a^	
Low-medium education	1,144 (29.0%)
Higher education	2,799 (71.0%)
Professional status	
Unemployed	317 (8.0%)
Employed	1,607 (40.8%)
Retired	1,871 (47.5%)
Financial situation	
Unsatisfactory	129 (3.3%)
Satisfactory	808 (20.5%)
More than satisfactory	2,968 (75.3%)
Living alone	1,011 (25.6%)
Living area, population density	
Rural	701 (17.8%)
Urban <40.000	1,525 (38.7%)
Urban >40.000	1,714 (43.5%)
Subjective memory complaints and worries	572 (14.5%)
LIBRA-score^b^	−0.32 ± 2.2
LIBRA Risk factors	
Coronary heart disease	277/1,984 (14.0%)
Chronic renal disease	14/1,984 (0.7%)
Diabetes	133/1,984 (6.7%)
Obesity	325/1,984 (16.1%)
High cholesterol	461/1,984 (23.2%)
Hypertension	605/1,984 (30.5%)
Depressive feelings	193/1,984 (9.7%)
Physical inactivity	487/1,984 (24.5%)
Current smoking	90/1,984 (4.5%)
LIBRA Protective factors	
Alcohol (no or low/moderate)	1,159/1,984 (58.4%)
Healthy diet (Mediterranean)	1,365/1,984 (68.8%)
High cognitively active	1,728/1,984 (87.1%)
Past COVID-19-infection (yes)	252 (6.4%)
Fear of COVID-19-infection (yes)	1,248 (31.7%)

*Data are presented as n/N (%) were N is the total number of participants with available data or mean ± SD.*

*^a^Higher education represents higher professional education and university degrees and low-medium education completed primary school and/or lower/middle vocational education.*

*^b^LIBRA score was available for n = 1984, negative score represents more protective factors, and positive score more modifiable risk factors for cognitive decline.*

### Description of Changes in Modifiable Lifestyle Factors Related to Brain Health

**FIGURE 1:**
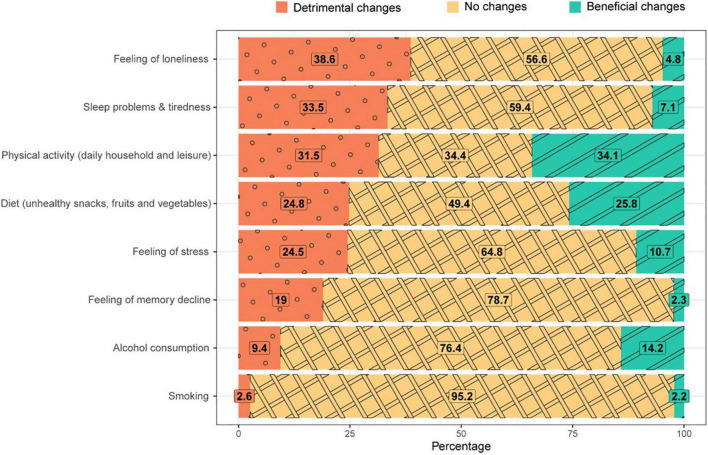
Self-reported changes in percentages across eight modifiable factors related to brain health after one year of COVID-19 restrictions (*n* = 3943).

Figure 1 illustrates the percentages of self-reported change on the eight modifiable factors related to cognitive decline. Most often reported detrimental changes were more feelings of loneliness (38.6%), an increase of sleep problems and feelings of tiredness (33.5%) and a decrease in the amount of physical activities (31.5%). As beneficial changes, participants most often reported an increase in physical activities (34.1%), healthy changes in diet (25.8%), and a decrease in alcohol consumption (14.2%). Substantial proportions of participants also reported no change with regards to each individual factor (34.4–95.2%).

**FIGURE 2:**
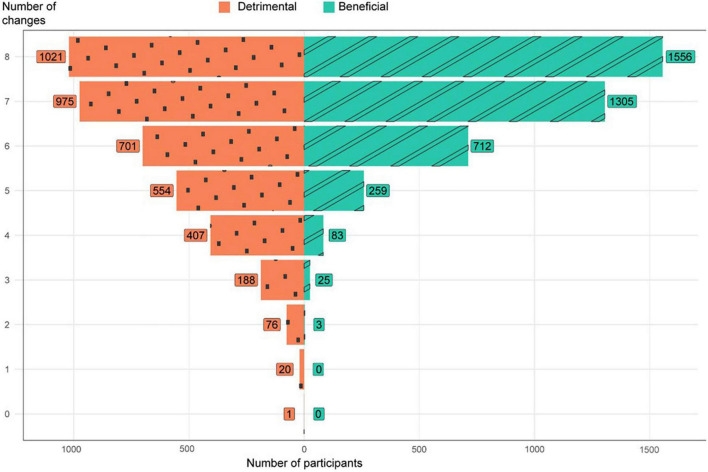
Frequencies of participants reporting number of detrimental or beneficial changes on modifiable factors related to brain health after one year of COVID-19 restrictions (*n* = 3943).

More than 7 in 10 respondents (*n* = 2852, 72.3%) reported at least one detrimental change (Figure 2), and 6 in 10 reported at least one healthy change (*n* = 2387, 60.5%); illustrating that many people showed both detrimental and beneficial lifestyle changes. When counting the number of reported factors that changed, 15.8% of participants (*n* = 622) reported detrimental changes on four or more factors compared to only 2.8% who reported (*n* = 111) four or more beneficial changes (Figure 2).

### Associations Between (Socio)demographics, LIBRA-Score and COVID-Characteristics, and Detrimental and Beneficial Changes

We identified associations of detrimental changes and beneficial lifestyle change related to brain health (Tables 2, 3). Similar relationships were found in Model 2 compared to Model 1 but slightly attenuated, only professional status was no longer statistically significant in Model 2. Participants with lower age (IRR = 0.99 [95% CI: 0.98–0.99]), female sex (IRR = 1.20 [1.11–1.30]), living alone (IRR = 1.20 [1.11–0.46]), presence of subjective memory complaints (IRR = 1.40 [1.28–1.51]), a current or past COVID-19 infection (IRR = 1.19 [1.06–1.34]) and a fear of a COVID-infection (IRR = 1.33 [1.25–1.42]) were more likely to report more detrimental changes compared to their reference groups. Those who were less satisfied with their income (compared to participants that were more than satisfied with their income; IRR = 1.38 [1.17–1.62]) and those living in an urban environment (compared to living in rural environment; IRR = 1.18 [1.08–1.29]) were also more likely to report more detrimental changes. When we evaluated the factors associated with beneficial changes (Table 3), we found that women (IRR = 1.16 [1.06–1.27]) and participants that reported fear of COVID-19 infection (IRR = 1.14 [1.06–1.24] were more likely to report beneficial changes. We found no other factors associated with beneficial changes (Table 3). As a *post hoc* analysis we conducted the multivariable analyses stratified by sex (see Supplementary Tables 3, 4), where living area, satisfactory financial situation (compared to a more than satisfactory financial situation), a current or past COVID-19 infection and high-risk for cognitive decline (measured by LIBRA) were associated with higher likelihood for reporting more detrimental lifestyle changes in women, but not in men.

**TABLE 2:** Univariable and multivariable analyses of the association of participant characteristics with multiple detrimental lifestyle changes.

	Model 1		Model 2	
	IRR [95% CI]	*p*-value	IRR [95% CI]	*p*-value
Age (years)^a^	**0.99 [0.98–0.99]**	**<0.0001**	**0.99 [0.98–0.99]**	**<0.0001**
Female^a^	**1.39 [1.29–1.49]**	**<0.0001**	**1.20 [1.11–1.30]**	**<0.0001**
Low-medium education	1.02 [0.96–1.09]	0.473	1.00 [0.93–1.07]	0.893
Professional status				
Unemployed	**1.19 [1.06–1.35]**	**0.004**	1.07 [0.94–1.22]	0.286
Employed	1.03 [0.94–1.13]	0.054	1.05 [0.96–1.16]	0.293
Retired	Ref		Ref	
Financial situation				
Unsatisfactory	**1.55 [1.35–1.79]**	**<0.0001**	**1.38 [1.17–1.62]**	**<0.0001**
Satisfactory	**1.26 [1.18–1.35]**	**<0.0001**	**1.21 [1.12–1.30]**	**<0.0001**
More than satisfactory	Ref		Ref	
Living alone (yes)	**1.24 [1.16–1.32]**	**<0.0001**	**1.20 [1.11–1.28]**	**<0.0001**
Living area, population density				
Rural	Ref		Ref	
Urban: small city <40.000	**1.17 [1.08–1.28]**	**<0.0001**	**1.13 [1.03–1.23]**	**0.011**
Urban: big city >40.000	**1.24 [1.14–1.34]**	**<0.0001**	**1.18 [1.08–1.29]**	**<0.0001**
Subjective memory complaints and worries	**1.49 [1.38–1.60]**	**<0.0001**	**1.40 [1.28–1.51]**	**<0.0001**
Current or past COVID-19 infection	**1.18 [1.05–1.32]**	**0.004**	**1.19 [1.06–1.34]**	**0.002**
Fear of COVID-19 infection	**1.36 [1.28–1.45]**	**<0.0001**	**1.33 [1.25–1.42]**	**<0.0001**
Health and lifestyle risk for cognitive decline (LIBRA score)^b^	**1.05 [1.03–1.07]**	**<0.0001**		**0.004**
Low-risk [<−1.6]	Ref		Ref	
Intermediate-risk [1.6 to 0.4]	1.08 [0.97–1.19]	0.166	1.07 [0.96–1.19]	0.250
High-risk [>0.4]	**1.24 [1.12–1.37]**	**<0.0001**	**1.15 [1.03–1.28]**	**0.011**

*CI, confidence interval; IRR, incidence rate ratio; ref, reference category. In bold are statistically significant. Model 1, univariable models corrected for sex and age; Model 2, multivariable analysis (n = 3274) where all predictors were entered simultaneously.*

*^a^Variables are not corrected. ^b^LIBRA score was analyzed as additional determinant in a subsample, Model 1 (n = 1981), univariable models corrected for sex and age; Model 2, multivariable analysis (n = 1697) where all predictors from model 1 were entered simultaneously.*

**TABLE 3:** Univariable and multivariable analyses of the association of participant characteristics with multiple beneficial lifestyle changes.

	Model 1		Model 2	
	IRR [95% CI]	*p*-value	IRR [95% CI]	*p*-value
Age (years)^a^	**1.38 [1.05–1.82]**	**0.026**	1.00 [1.00–1.00]	0.407
Female^a^	**1.14 [1.05–1.23]**	**0.002**	**1.16 [1.06–1.27]**	**0.001**
Low-medium education	0.96 [0.89–1.03]	0.245	0.98 [0.90–1.06]	0.571
Professional status				
Unemployed	0.94 [0.81–1.09]	0.430	0.91 [0.77–1.07]	0.244
Employed	1.06 [0.95–1.17]	0.308	1.06 [0.94–1.18]	0.344
Retired	Ref		Ref	
Financial situation				
Unsatisfactory	1.00 [0.83–1.20]	0.968	1.12 [0.91–1.37]	0.291
Satisfactory	0.95 [0.87–1.03]	0.192	0.93 [0.84–1.02]	0.119
More than satisfactory	Ref		Ref	
Living alone (yes)	0.93 [0.86–1.01]	0.086	0.93 [0.85–1.01]	0.088
Living area, population density				
Rural	Ref		Ref	
Urban: small city <40.000	1.00 [0.91–1.16]	0.944	1.01 [0.91–1.12]	0.891
Urban: big city >40.000	1.03 [0.97–1.16]	0.222	1.07 [0.96–1.19]	0.215
Subjective memory complaints and worries	0.98 [0.89–1.08]	0.698	1.00 [0.90–1.12]	0.938
Current or past COVID-19 infection	0.94 [0.82–1.08]	0.403	0.97 [0.84–1.12]	0.636
Fear of COVID-19 infection	**1.13 [1.05–1.22]**	**0.001**	**1.14 [1.06–1.24]**	**0.001**
Health and lifestyle risk for cognitive decline (LIBRA score)^b^	1.19 [1.07–1.33]	0.077	0.98 [0.96–1.01]	0.350
Low-risk [<−1.6]	Ref		Ref	
Intermediate-risk [−1.6 to 0.4]	**0.87 [0.77–0.97]**	**0.014**	**0.85 [0.75–0.96]**	**0.012**
High-risk [>0.4]	**0.89 [0.79–0.99]**	**0.039**	0.89 [0.78–1.00]	0.056

*CI, confidence interval; IRR, incidence rate ratio; ref, reference category. In bold are statistically significant. Model 1, univariable models corrected for sex and age; Model 2, multivariable analysis (n = 3274) where all predictors were entered simultaneously.*

*^a^Variables are not corrected. ^b^LIBRA score was analyzed as additional determinant in a subsample, Model 1 (n = 1981), univariable models corrected for sex and age; Model 2, multivariable analysis (n = 1697) where all predictors from model 1 were entered simultaneously.*

Participants with high-risk for cognitive decline (in the upper tertile of LIBRA scores) were more likely to report a higher number of detrimental changes (IRR = 1.15 [1.03–1.28]) compared to participants with low-risk for cognitive decline (in the lowest tertile of LIBRA scores). For beneficial changes, participants with intermediate-risk on the LIBRA score reported less beneficial changes (IRR = 0.85 [0.75–0.96]). For participants with high-risk, we identified similar effect on trend level (IRR = 0.89 [0.78–1.00], *p* = 0.056).

Finally, when we conducted a sensitivity analysis, excluding participants with a current or past COVID-19 infection (*n* = 252) results remained essentially unchanged for detrimental changes (Supplementary Tables 5, 6). For beneficial changes, we additionally found that the likelihood for reporting more beneficial changes decreased (IRR = 0.89 [0.81–0.98]) for participants living alone.

## Discussion

After one year of COVID-19 pandemic and related restrictions in Netherlands, 72.3% respondents aged ≥50 reported at least one detrimental lifestyle change unfavorable for their brain health, while 60.5% reported at least one beneficial change. The most often reported detrimental changes were more feelings of loneliness (38.6%), more sleep problems (33.5%), and less physical activity (31.5%). Most frequently reported beneficial changes were more physical activity (34.1%), healthier dietary habits (25.8%), and less alcohol consumption (14.2%). Lower age, female sex, living alone and in urban environments, presence of subjective memory complaints, a current or past COVID-19 infection and fear of getting infected was associated with experiencing more detrimental changes in lifestyle. In contrast to our expectations, we found only few associations of social determinants with beneficial changes including female sex and fear of COVID-19 infection.

Interestingly, women report both more detrimental and more beneficial lifestyle changes compared to men. In addition, women in our sample were more often employed compared to men (45% compared to 32%). Possible reasons why COVID-19 pandemic creates greater challenges especially for women may be that on average, women earn less money (30), and are more likely to work in healthcare (31), all of which could be expected to impose a greater burden during a pandemic and thereby influencing their ability to maintain a healthy lifestyle (32). On the other hand, our results also suggest that women seemingly do have the ability to improve their lifestyle during the pandemic. Alternatively, women might be likely to observe and report change. In order to draw conclusion about the motives of women changing their lifestyles, more research and qualitative studies are needed.

Fear of getting a COVID-19 infection was associated with increased likelihood of reporting more detrimental lifestyle and more beneficial lifestyle changes. Campaigns during COVID-19 pandemic primarily focus on fear appeal and solidarity with more vulnerable citizens. Previous research has reported that campaigns using fear appeal (33) and stressful life events with subsequent effects (34) can positively influence attitude, intentions, and behaviors. On the other hand, effects of prolonged fear may differ, and in a context of public health and economic uncertainty such the COVID-19 pandemic, fear appeal may also induce negative side effects among vulnerable individuals (21). Additionally, a cross-national survey conducted suggested that controlling fear response would help to improve health outcomes, however, they observed differences across countries emphasizing possible social, cultural and economic influences on fear response and health outcomes (16).

In line with other studies (12, 21), younger elderly are more prone to report higher number of detrimental lifestyle changes during the pandemic, which might be due to persistent difficulty of balancing work (21) and less work-related commute and social interactions, possibly causing stress and social isolation. Unhealthy lifestyle has been related to social determinants like education, income and physical environment (9, 12, 35, 36). More specifically, a previous study showed that those with lower social economic status had higher risk for dementia due to accumulation of modifiable health and lifestyle risk factors (measured by LIBRA) (37). This is in line with our findings, as we found that participants with lower income were more likely to report multiple detrimental changes. Participants with more modifiable health and lifestyle risk factors (measured by LIBRA) report a higher number of detrimental lifestyle changes during the COVID-19 pandemic, pointing at a potential further risk of cognitive decline and dementia. These results contribute to the notion the COVID-19 pandemic may amplify health inequalities in brain health and dementia risk (38, 39).

In a relatively young elderly sample (mean age 66 ± 8 years), we found that roughly a quarter reported more feelings of loneliness. Additional *post hoc* analysis showed that living alone was associated with reporting more often feelings of loneliness (data not shown). Also, those living in urban environments (compared to living in rural environment) reported more detrimental lifestyle changes. Therefore living conditions seem to be risk factors for more detrimental lifestyle changes, as in line with previous studies (9).

Moreover, pre-existent subjective cognitive complaints were associated with more detrimental changes in modifiable factors related to cognitive decline, suggesting that those at risk of dementia may further increase their dementia risk during the pandemic (40). In line with this observation, a previous Italian study of older adults with pre-existing cognitive problems (subjective cognitive decline or mild cognitive impairment) reported more lifestyle behaviors that are potentially harmful for future cognitive decline (41). These results emphasize the potential of lifestyle interventions, especially in those who are at-risk for cognitive decline and dementia.

In general, comparing impact of the COVID-19 pandemic on lifestyle across countries imposes challenges, since COVID-19 restrictions may differ over time, and different social and cultural aspects apply. Comparing our results to those of a study within Dutch individuals during the first wave (21), unhealthy changes were slightly more often reported by our study participants after one year of COVID-19-restrictions. Since restrictions were more severe during the second wave in October 2020 and February 2021 (closing of schools, non-essential shops and cultural- and sport facilities, and a curfew) compared to the first wave from March 2020 to July 2020, together with a prolonged exposure to the pandemic and related restrictions in our study, this may have influenced moral and motivation to improve or maintain a healthy lifestyle. When comparing results with findings of the World-Wide-FINGERS-SARS-CoV-2 survey carried out in a Finish population at risk for cognitive decline (FINGER participants) during the first wave, some proportions are similar for detrimental and beneficial changes (physical activity, dietary habits, memory complaints) while others seem to be higher in our study (feelings of loneliness, sleep problems, alcohol intake, and smoking) (9). This may imply that some factors deteriorate with longer duration of the pandemic, while others factors remain relatively stable or happened in a similar extent.

This study had several limitations; the assessment of lifestyle behavior was done online, relied on self-report surveys and retrospective assessment, and therefore not objective or validated. Additionally, past or current COVID-19 infection was not validated with clinical records. The survey was widely distributed among a population sample, the voluntary approach may have led to a selection bias and consequently detrimental lifestyle changes may have been over reported. However, the online nature of the survey and the use of a nation-wide registry (24) allowed us to include a large sample of participants. Another limitation of this sample was that it was mainly female, highly educated and had a higher income compared to the general Dutch population, hence findings on average percentages reporting no, mainly beneficial or mainly detrimental change might not generalize. Due to our exploratory and cross-sectional approach, replication, follow-up and additional qualitative research is required to further explore the impact of COVID-19 pandemic and associations of social determinants with lifestyle changes. Lastly, The World-Wide-FINGERS-SARS-CoV-2 survey was not validated and a subset of questions was selected. Complete results of the survey will be combined with results of the same surveys conducted in other countries, and will be published elsewhere.

Among the strengths was that the study was conducted after a period of one year of COVID-19 restrictions in the Netherlands, thus capturing medium-long term health consequences rather than the initial response to the COVID-restrictions. Enduring unhealthy changes in lifestyle will be more detrimental, thus making our results more imperative. Furthermore, we focused on both detrimental and beneficial lifestyle changes. Additionally, to estimate the future risk of cognitive decline we used the LIBRA score, which is a well-validated measurement for predicting cognitive decline and higher dementia risk in various general population and patient-studies (42). Further, LIBRA focuses solely on modifiable risk factors, making participants with higher LIBRA scores especially susceptible for lifestyle changes and altering their future cognitive decline.

It has been suggested that the COVID-19 pandemic provides an opportunity for healthcare professionals to promote lifestyle change (43, 44). When regarding dementia risk, the general population is mostly unaware of its relation with lifestyle (45, 46). Our results show that the COVID-19 pandemic creates a window of opportunity for lifestyle intervention for the prevention of accelerated cognitive decline, especially for a specific population who seems to be more vulnerable for the detrimental changes in modifiable risk factors related to brain health during the COVID-19 pandemic. Therefore, providing evidence and motivation for health initiatives to (i) create awareness among health care professionals and the population about the relationship between lifestyle and dementia risk (e.g., by encouraging multi-domain lifestyle interventions to prevent cognitive decline), (ii) create (targeted) educational tools to promote/maintain a brain-healthy lifestyle, and (iii) implement health surveillance to monitor lifestyle changes during lockdowns, preferably using online technologies to fit to the current health care situation in the COVID-19 pandemic. As there has been little to no cost-benefit analysis prior to the introduction of governmental measures and restrictions during the COVID-19 pandemic, actual costs of health outcomes and lifestyle changes may become more apparent in the future. Additionally, for future lockdowns preventative strategies such as promoting a healthy lifestyle are important alternative options to fight the spread of disease or reduce the risk of severe complications or hospitalization due an infection, as it is a boost for one’s individual natural resistance. Furthermore, from a broader public health perspective, preventive strategies are also very important to consider to reduce an individuals’ risk of other non-communicable diseases for instance vascular and metabolic diseases.

## Data Availability Statement

The datasets presented in this article are not readily available because of ethical reasons. Data contains large amount of sensitive information and public data deposition may pose privacy concerns. Further, it is not possible as this dataset is part of an WW-FINGERS initiative. The study participants provided online informed consent for local analysis at Alzheimer Center Amsterdam and only to share pseudonymized data with WW-FINGERS partners. Requests to access the datasets should be directed to MZ, m.zwan@amsterdamumc.nl.

## Ethics Statement

The studies involving human participants were reviewed and approved by the Medisch Ethische Toetsingscommissie VU Medisch Centrum. Written informed consent for participation was not required for this study in accordance with the National Legislation and the Institutional Requirements.

## Author Contributions

MZ and WF revised the study objectives. FM and MK provided the WW-FINGERS survey. LW and EB translated the survey and were responsible for data collection. MZ and LW interpreted the results. LW conducted the data analysis and wrote the manuscript. All authors critically revised the manuscript and contributed to the article and approved the submitted version.

## Conflict of Interest

SS provided consultancy services (Biogen, Boehringer and Toyama) and received license fees (Green Valley, VtV Therapeutics, Alzheon, Vivoryon, Roche); all funds were paid to the institution. The remaining authors declare that the research was conducted in the absence of any commercial or financial relationships that could be construed as a potential conflict of interest.

## Publisher’s Note

All claims expressed in this article are solely those of the authors and do not necessarily represent those of their affiliated organizations, or those of the publisher, the editors and the reviewers. Any product that may be evaluated in this article, or claim that may be made by its manufacturer, is not guaranteed or endorsed by the publisher.

## Footnotes

1
https://wwfingers.com
