# Supplementary material for: Changes in Brain-Health Related Modifiable Risk Factors in Older Adults After One Year of COVID-19-Restrictions
Source: Front Psychiatry. 2022 Jun 2;13:877460. doi: 10.3389/fpsyt.2022.877460 (PMC9201112; doi:10.3389/fpsyt.2022.877460)
Supplement: Supplementary file 1 [file Data_Sheet_1.PDF]

Supplementary table 1: Overview questions for each modifiable lifestyle factor related to brain health.

Brain-health related factors	Question	Answer including scoring
FEELING OF LONELINESS	Has the COVID-19 pandemic or restrictions associated with it affected your daily life?<br>Please check “Does not concern me” if a matter on the list is not relevant to your life at all.<br>Experience of loneliness	a. Yes, decreased a lot (+1)<br>b. Yes, decreased a little (+1)<br>c. No change (0)<br>d. Yes, increased a little (-1)<br>e. Yes, increased a lot (-1)<br>f. Does not concern me (0)
SLEEP	Has the COVID-19 pandemic or restrictions associated with it affected your daily life?<br>Please check “Does not concern me” if a matter on the list is not relevant to your life at all.<br>Sleep problems	a. Yes, decreased a lot (+1)<br>b. Yes, decreased a little (+1)<br>c. No change (0)<br>d. Yes, increased a little (-1)<br>e. Yes, increased a lot (-1)<br>f. Does not concern me (0)
SLEEP	In times of Covid-19, are you feeling more tired?	a. Yes (-1)<br>b. No, less tired (+1)<br>c. I feel the same (0)<br>d. I do not know (0)<br>e. I am never tired (0)
PHYSICAL ACTIVITY	Has the COVID-19 pandemic or restrictions associated with it affected your daily life? Please check “Does not concern me” if a matter on the list is not relevant to your life at all.<br>○ Daily physical activity (e.g., time spent walking or cycling as part of commuting, gardening, doing housework)	a. Yes, decreased a lot (-1)<br>b. Yes, decreased a little (-1)<br>c. No change (0)<br>d. Yes, increased a little (+1)<br>e. Yes, increased a lot (+1)<br>f. Does not concern me (0)
PHYSICAL ACTIVITY	How often did you engage in leisure time physical activity/planned exercise before the outbreak of the COVID-19 pandemic in your country? Count activities that last at least 20 minutes and that cause at least mild sweating and breathlessness. Also home-based physical activity is included if you are out of breath and sweating.<br><br>COMPARED TO:<br>How often have you engaged in leisure time physical activity/planned exercise during the COVID-19 pandemic in your country? Count activities that last at least 20 minutes and that cause at least mild sweating and breathlessness. Also home-based physical activity is included if you are out of breath and sweating.	a. Never<br>b. Less than once a month<br>c. 1-2 times a month<br>d. About once a week<br>e. 2-3 times a week<br>f. 4-5 times a week<br>g. Approximately every day<br>h. I cannot due to injury or illness<br>_________________________<br>a. Never<br>b. Less than once a month<br>c. 1-2 times a month<br>d. About once a week<br>e. 2-3 times a week<br>f. 4-5 times a week<br>g. Approximately every day<br>h. I cannot due to injury or illness

DIET	Has the COVID-19 pandemic or restrictions associated with it affected your daily life?<br>Please check "Does not concern me" if a matter on the list is not relevant to your life at all.<br>o Vegetable consumption (also raw vegetables but not potato)	a. Yes, decreased a lot (-1)<br>b. Yes, decreased a little (-1)<br>c. No change (0)<br>d. Yes, increased a little (+1)<br>e. Yes, increased a lot (+1)<br>f. Does not concern me (0)
DIET	Has the COVID-19 pandemic or restrictions associated with it affected your daily life?<br>Please check "Does not concern me" if a matter on the list is not relevant to your life at all.<br>o Fruit or berries consumption	a. Yes, decreased a lot (-1)<br>b. Yes, decreased a little (-1)<br>c. No change (0)<br>d. Yes, increased a little (+1)<br>e. Yes, increased a lot (+1)<br>f. Does not concern me (0)
DIET	Has the COVID-19 pandemic or restrictions associated with it affected your daily life?<br>Please check "Does not concern me" if a matter on the list is not relevant to your life at all.<br>o Snacking (sweets, chocolate, softdrinks, chips, crisps, etc.)	a. Yes, decreased a lot (+1)<br>b. Yes, decreased a little (+1)<br>c. No change (0)<br>d. Yes, increased a little (-1)<br>e. Yes, increased a lot (-1)<br>f. Does not concern me (0)
FEELING OF STRESS	In times of Covid-19, are you feeling more stressed?	a. Yes (-1)<br>b. No, less stress (+1)<br>c. I feel the same (0)<br>d. I do not know (0)<br>e. I am never stressed (0)
MEMORY COMPLAINTS	Do you think there have been any significant changes in your memory since the start of the COVID-19 pandemic in your country?	a. Yes, my memory is clearly becoming worse (-1)<br>b. Yes, my memory is slightly becoming worse (-1)<br>c. No, my memory has remained the same (0)<br>d. Yes, my memory is slightly becoming better (+1)<br>e. Yes, my memory is clearly becoming better (+1)<br>f. I do not know (0)
ALCOHOL CONSUMPTION	Has the COVID-19 pandemic or restrictions associated with it affected your daily life?<br>Please check "Does not concern me" if a matter on the list is not relevant to your life at all.<br>o Alcohol use	a. Yes, decreased a lot (+1)<br>b. Yes, decreased a little (+1)<br>c. No change (0)<br>d. Yes, increased a little (-1)<br>e. Yes, increased a lot (-1)<br>f. Does not concern me (0)
SMOKING	Has the COVID-19 pandemic or restrictions associated with it affected your daily life?<br>Please check "Does not concern me" if a matter on the list is not relevant to your life at all.<br>o Smoking	a. Yes, decreased a lot (+1)<br>b. Yes, decreased a little (+1)<br>c. No change (0)<br>d. Yes, increased a little (-1)<br>e. Yes, increased a lot (-1)<br>Does not concern me (0)

Supplementary table 2. Individual's health and lifestyle prevention potential before the COVID-19 pandemic defined by "Lifestyle for Brain health"(LIBRA).

Factor	Question	Answer including scoring
**Risk**		
Heart disease	Has your doctor ever told you that you have a heart-or blood vessel condition (including heart attack, angina (chest pain), heart failure, stroke or TIA's)?	Yes (+1.0)<br>No (0)<br>I don't know (0)
Physical inactivity	Do you generally consider yourself as an active person, who regularly performs exercise in which you start to sweat lightly?	Yes (0)<br>A little (0)<br>No (+1.1)
Chronic kidney disease	Has your doctor ever told you that you have chronic kidney disease?	Yes (+1.1)<br>No (0)<br>I don't know (0
Diabetes	Has your doctor ever told you that you have diabetes?	Yes (+1.3)<br>No (0)<br>I don't know (0)
Cholesterol	Has your doctor ever told you that your cholesterol is	Yes (+1.4)<br>No (0)<br>I don't know (0)
Smoking	Do you smoke?	Yes (+1.5)<br>No (0)
Obesity	How long are you (in centimeters)?<br>How much do you weigh? Round off on whole kilograms. Compute BMI = weight (kg) / length (m2)	BMI ≥ 30 (+1.6)<br>BMI <30 (0)
Hypertension	Has your doctor ever told you that you have high blood pressure?	Yes (+1.6)<br>No (0)<br>I don't know (0)
Depression	Generally, I feel cheerful and consider my life worth living.	Agree (0)<br>Don't agree (+2.1)
**Protective**		
Low-to-moderate alcohol consumption*	What is your daily, average alcohol consumption?	don't drink alcohol (-1.0)<br>Up to 1 glass a day (-1.0)<br>More than 1 glass a day (0)
Healthy diet/Mediterranean diet	Someone who adheres to the Mediterranean diet regularly eats fish, vegetables, legumes, (olive) oil, pasta and sometimes drinks a glass of red wine. Do you recognize your own diet in this?	Yes (-1.7)<br>A little (0)<br>No (0)
High cognitive activity	Do you generally consider yourself as someone who is mentally active, such as by learning new things, making music, practicing hobbies?	Yes (-3.2)<br>A little (0)<br>No (0)

* Cut-off for low-to-moderate alcohol consumption based on Dutch Dietary Guidelines; Health Council of the Netherlands (2015) *Dutch dietary guidelines 2015*, Health Council of the Netherlands, The Hague

Supplementary Table 3. Sensitivity analysis stratified by sex. Multivariable analysis of the association of participant characteristics with detrimental lifestyle changes stratified by sex.

	Multivariable Females (n = 2988)		Multivariable Males (n=948)	
	IRR [95% CI]	p-value	IRR [95% CI]	p-value
Age (years) [a]	**0.98 [0.98 – 0.99]**	**<.0001**	1.00 [0.99 – 1.01]	.469
Lower education	1.02 [0.95 – 1.01]	.595	0.88 [0.74 – 1.04]	.126
Professional status				
Unemployed	1.05 [0.91 – 1.21]	.505	1.16 [0.84 – 1.60]	.362
Employed	1.03 [0.93 – 1.15]	.575	1.14 [0.93 – 1.39]	.224
Retired	ref		ref	
Financial situation:				
Unsatisfactory	**1.35 [1.13 – 1.62]**	**.001**	**1.43 [1.01 – 2.02]**	**.045**
Satisfactory	**1.25 [1.15 – 1.36]**	**<.0001**	1.01 [0.93 – 1.23]	.911
More than satisfactory	ref		ref	
Living alone (yes)	**1.17 [1.08 – 1.27]**	**<.0001**	**1.37 [1.15 – 1.93]**	**<.0001**
Living area, population density				
Rural	ref		ref	
Urban: small city <40.000	**1.17 [1.06 – 1.30]**	**.002**	0.97 [0.79 – 1.20]	.792
Urban: big city >40.000	**1.20 [1.09 – 1.33]**	**<.0001**	1.10 [0.90 – 1.36]	.343
Subjective memory complaints and worries	**1.32 [1.20 – 1.15]**	**<.0001**	**1.72 [1.44 – 2.04]**	**<.0001**
Current or past COVID-19 infection	**1.17 [1.03 – 1.33]**	**.017**	1.27 [0.98 -1.64]	.068
Fear of COVID-19 infection	**1.33 [1.24 – 1. 43]**	**<.0001**	**1.34 [1.16 – 1.55]**	**<.0001**
Health and lifestyle risk for cognitive decline (LIBRA score)[b]	**1.03 [1.01 – 1.05]**	**.009**	1.02 [0.98 – 1.07]	.307
Low-risk [< -1.6]	ref		ref	
Intermediate-risk [-1.6 to 0.4]	1.07 [0.94 – 1.21]	.296	1.05 [0.83 – 1.31]	.702
High-risk [> 0.4]	**1.18 [1.04 – 1.34]**	**.008**	1.07 [0.86 – 1.33]	.564

Abbreviation: CI Confidence Interval; RR rate ratio; ref reference category. In bold are statistically significant. Multivariable analysis where all predictors were entered simultaneously.
[a] Variables are not corrected. [b]LIBRA score was analyzed as additional determinant in a subsample

Supplementary Table 4. Sensitivity analysis stratified by sex. Multivariable analyses of the association of participant characteristics with beneficial lifestyle changes stratified by sex.

	Multivariable Females (n = 2988)		Multivariable Males (n=948)	
	IRR [95% CI]	p-value	IRR [95% CI]	p-value
Age (years) [a]	1.00 [0.99 – 1.01]	.489	1.00 [0.99 – 1.01]	.781
Lower education	0.97 [0.88 – 1.06]	.479	1.01 [0.84 – 1.21]	.927
Professional status				
Unemployed	0.87 [0.72 – 1.04]	.127	1.34 [0.78 – 1.65]	.512
Employed	1.01 [0.89 – 1.16]	.853	1.22 [0.98 – 1.52]	.071
Retired	ref		ref	
Financial situation:				
Unsatisfactory	1.24 [0.99 – 1.55]	.065	0.69 [0.41 – 1.17]	.168
Satisfactory	0.93 [0.83 – 1.03]	.167	0.92 [0.74 – 1.14]	.448
More than satisfactory	ref		ref	
Living alone (yes)	0.92 [0.84 – 1.02]	.116	0.90 [0.72 – 1.12]	.339
Living area, population density				
Rural	ref		ref	
Urban: small city <40.000	0.99 [0.88 – 1.12]	.860	1.09 [0.86 – 1.38]	.484
Urban: big city >40.000	1.04 [0.92 – 1.17]	.519	1.20 [0.95 – 1.51]	.126
Subjective memory complaints and worries	0.99 [0.88 – 1.12]	.860	1.06 [0.86 – 1.31]	.598
Current or past COVID-19 infection	0.94 [0.80 – 1.12]	.496	1.06 [0.79 – 1.41]	.700
Fear of COVID-19 infection	**1.14 [1.04 – 1.24]**	**.004**	**1.17 [1.00 – 1.38]**	**.050**
Health and lifestyle risk for cognitive decline (LIBRA score)[b]	**0.97 [0.95 – 1.00]**	**.050**	1.01 [0.96 – 1.06]	.722
Low-risk [< -1.6]	ref		ref	
Intermediate-risk [-1.6 to 0.4]	**0.85 [0.74 – 0.98]**	**.026**	0.87 [0.68 – 1.12]	.289
High-risk [> 0.4]	**0.84 [0.72 – 0.97]**	**.018**	1.03 [0.82 – 1.30]	.807

Abbreviation: CI Confidence Interval; RR rate ratio; ref reference category. In bold are statistically significant. Multivariable analysis where all predictors were entered simultaneously.
[a] Variables are not corrected. [b]LIBRA score was analyzed as additional determinant in a subsample

Supplementary Table 5. Sensitivity analysis. Univariable and multivariable analyses of the association of participant characteristics with detrimental lifestyle changes in participants with no current or past COVID-19 infection (n=3481).

	Model 1		*Model 2*	
	IRR [95% CI]	p-value	IRR [95% CI]	p-value
Age (years) [a]	**0.99 [0.98 – 0.99]**	**<.0001**	**0.99 [0.98 – 1.00]**	**<.0001**
Female [a]	**1.36 [1.26 – 1.47]**	**<.0001**	**1.21 [1.12 – 1.32]**	**<.0001**
Lower education	1.02 [0.95 – 1.10]	.536	1.00 [0.92 – 1.07]	0.899
Professional status				
Unemployed	**1.17 [1.03 – 1.33]**	**.020**	1.05 [0.91 – 1.20]	0.519
Employed	1.00 [0.91 – 1.11]	.940	1.05 [0.95 – 1.17]	0.317
Retired	ref		ref	
Financial situation:				
Unsatisfactory	**1.54 [1.31 – 1.81]**	**<.0001**	**1.41 [1.18 – 1.67]**	**<.0001**
Satisfactory	**1.24 [1.15 – 1.33]**	**<.0001**	**1.19 [1.10 – 1.29]**	**<.0001**
More than satisfactory	**ref**		ref	
Living alone (yes)	**1.24 [1.16 – 1.33]**	**<.0001**	**1.21 [1.12 – 1.29]**	**<.0001**
Living area, population density				
Rural	ref		ref	
Urban: small city <40.000	**1.18 [1.08 – 1.29]**	**<.0001**	**1.15 [1.04 – 1.27]**	**.005**
Urban: big city >40.000	**1.25 [1.15 – 1.37]**	**<.0001**	**1.20 [1.09 – 1.32]**	**<.0001**
Subjective memory complaints and worries	**1.48 [1.37 – 1.61]**	**<.0001**	**1.39 [1.27 – 1.51]**	**<.0001**
Current or past COVID-19 infection	NA	**-**	NA	**-**
Fear of COVID-19 infection	**1.36 [1.27 – 1.46]**	**<.0001**	**1.34 [1.25 -1.43]**	**<.0001**
Health and lifestyle risk for cognitive decline (LIBRA score)[b]	**1.06 [1.03 – 1.08]**	**<.0001**	**1.03 [1.01 – 1.06]**	**.002**
Low-risk [< -1.6]	**ref**		**ref**	
Intermediate-risk [-1.6 to 0.4]	1.04 [0.94 – 1.18]	.358	1.05 [0.93 – 1.18]	.431
High [> 0.4]	**1.25 [1.12 – 1.40]**	**<.0001**	**1.17 [1.04 – 1.31]**	**.008**

Abbreviation: CI Confidence Interval; IRR incidence rate ratio; NA not applicable; ref reference category. In bold are statistically significant. Model 1: univariable models corrected for sex and age. Model 2: multivariable analysis (n=3274) where all predictors were entered simultaneously. [a] Variables are not corrected. [b]LIRBA score was analyzed as additional determinant in a subsample, Model 1 (n=1981): univariable models corrected for sex and age, Model 2: multivariable analysis (n=1697) where all predictors from model 1 were entered simultaneously.

Supplementary Table 6. Sensitivity analysis. Univariable and multivariable analyses of the association of participant characteristics with beneficial lifestyle changes in participants with no current or past COVID-19 infection (n=3481).

	Model 1		*Model 2*	
	IRR [95% CI]	p-value	IRR [95% CI]	p-value
Age (years) [a]	**1.00 [1.00-1.00]**	**.011**	1.00 [0.99 – 1.01]	.701
Female [a]	**1.17 [1.07 – 1.27]**	**<.0001**	**1.18 [1.07 – 1.30]**	**.001**
Lower education	0.97 [0.89 – 1.04]	.379	0.99 [0.91 – 1.08]	.774
Professional status				
Unemployed	0.93[0.80 – 1.09]	.375	0.92 [0.78 – 1.09]	.337
Employed	1.07 [0.96 – 1.20]	.211	1.07 [0.95 – 1.20]	.261
Retired	ref		ref	
Financial situation:				
Unsatisfactory	1.01 [0.82 – 1.23]	.945	1.13 [091 – 1.40]	.264
Satisfactory	0.92 [0.85 – 1.01]	.082	0.93 [0.85 – 1.03]	.174
More than satisfactory	ref		ref	
Living alone (yes)	**0.90 [0.82 – 0.98]**	**.010**	**0.89 [0.81 – 0.98]**	**.014**
Living area, population density				
Rural	ref		ref	
Urban: small city <40.000	1.00 [0.90 – 1.11]	.996	0.99 [0.88 – 1.10]	.817
Urban: big city >40.000	1.07 [0.97 – 1.19]	.165	1.07 [0.96 – 1.20]	.197
Subjective memory complaints and worries	0.97 [0.87 – 1.07]	.533	1.02 [0.91 – 1.14]	.743
Current or past COVID-19 infection	NA	-	NA	-
Fear of COVID-19 infection	**1.17 [1.08 – 1.26]**	**<.0001**	**1.16 [1.08 – 1.26]**	**<.0001**
Health and lifestyle risk for cognitive decline (LIBRA score)[b]	0.98 [0.96 -1.00]	.112	0.98 [0.96 – 1.01]	.183
Low-risk [< -1.6]	ref		ref	
Intermediate-risk [-1.6 to 0.4]	**0.88 [0.78 – 0.99]**	**.039**	**0.86 [0.76 – 0.98]**	**.024**
High-risk [> 0.4]	0.89 [0.79 – 1.00]	.057	0.89 [0.78 – 1.01]	.072

Abbreviation: CI Confidence Interval; IRR incidence rate ratio; NA not applicable ref reference category. In bold are statistically significant. Model 1: univariable models corrected for sex and age. Model 2: multivariable analysis (n=3274) where all predictors were entered simultaneously. [a] Variables are not corrected. [b]LIRBA score was analyzed as additional determinant in a subsample, Model 1 (n=1981): univariable models corrected for sex and age, Model 2: multivariable analysis (n=1697) where all predictors from model 1 were entered simultaneously.
